# Acoustic Insulation Characteristics and Optimal Design of Membrane-Type Metamaterials Loaded with Asymmetric Mass Blocks

**DOI:** 10.3390/ma16031308

**Published:** 2023-02-03

**Authors:** Renjie Jiang, Geman Shi, Chengmao Huang, Weiguang Zheng, Shande Li

**Affiliations:** 1 State Key Laboratory of Digital Manufacturing Equipment and Technology, School of Mechanical Science and Engineering, Huazhong University of Science and Technology, Wuhan 430074, China; 2 School of Mechanical and Automotive Engineering, Guangxi University of Science and Technology, Liuzhou 545006, China; 3 Hubei Innovation Center of Mobile Emergency Equipment Manufacturing, Hubei Institute of Specialty Vehicle, Suizhou 441300, China

**Keywords:** membrane-type acoustic metamaterials, sound transmission loss, asymmetric mass

## Abstract

Membrane-type acoustic metamaterials (MAMs) are the focus of the current research due to their lightweight, small size, and good low-frequency sound insulation performance. However, there exists difficulties for extensive application because of the narrow sound insulation band. In order to achieve broadband sound isolation under the premise of lightweight, a novel MAM with asymmetric rings is firstly proposed in this paper. The sound transmission loss (STL) of this MAM is calculated by an analytical method and is verified by the finite element model. The different properties of the membrane when it is loaded with one, two, or four mass blocks are analyzed. The comparison with the traditional MAM proves the superior performance of this novel MAM. Moreover, by discussing the influence of the eccentricity and distribution position of the masses on the results, the tunability of the sound insulation performance of this MAM is proven. Finally, the Isight platform is used to optimize the MAM to further improve the broadband sound insulation performance: the average STL of the MAM is improved by 15.7%, the bandwidth above 30 dB is improved by 11.5%, and the mass density is reduced by 30.01%.

## 1. Introduction

Noise pollution has been a serious problem for many years, and sound insulation as an important noise reduction method has been studied by many scholars. In order to effectively block low-frequency noise, it is necessary to design a thick and heavy sound insulation structure. Therefore, how to achieve high-performance sound insulation under the premise of lightweight has become a research focus [1,2]. In recent years, the emergence of acoustic metamaterials has opened up a new path for low-frequency noise insulation [3,4,5,6,7,8,9,10]. Acoustic metamaterials are artificially constructed periodic structures that are different from traditional materials in nature and can break the law of mass. Membrane-type acoustic metamaterials (MAMs) are typical sound-insulating metamaterials which can generate good sound-insulating peaks in a specific frequency band with a compact and lightweight structure, and they have attracted more attention in recent years [11,12,13,14,15,16,17].

Theoretical research on calculating the transmission loss of MAMs has achieved certain results. Zhang et al. [18] firstly proposed an analytical model, which simplifies the mass block into the form of additional surface density loaded on the membrane and solves the vibration behavior of the membrane via the Galerkin method. Later, Chen et al. [19] proposed a point-matching method, which discretizes the force of the mass block into a series of point forces to change the solved equation into a nonlinear form and makes the result more accurate. On the basis of Chen’s method, Langfeldt et al. [20] solved the eigenfrequency and eigenvector of the coupled system by obtaining a generalized linear eigenvalue problem, and then calculated the sound transmission loss with the mass law to greatly reduce the calculation time. Moreover, this method can be applied to MAMs with any shape and any number of masses. Li et al. [21] simplified the description of arbitrarily shaped mass blocks using a conformal mapping method, which further reduced the calculation time of the STL.

However, MAMs also have challenges in low-frequency sound insulation. MAMs exhibit extremely high performance near resonance frequencies due to local resonance properties but are inefficient at other frequencies. In order to widen its sound insulation frequency band, many scholars have improved the design of the mass block structure. Naify et al. [22] studied MAMs with multiple ring masses and found that the sound transmission loss bandwidth and the number of peaks change with different mass, radii, and distributions of rings. Zhou et al. [23] designed a symmetrical cross-shaped mass block structure that is easy to achieve dynamic balance according to the low-order vibration characteristics and realized multi-state continuous sound insulation peaks. On the basis of Zhou’s model, Huang et al. [24] proposed a new MAM with petal-like rings, which achieved a similar sound insulation effect under lighter mass conditions. Lu et al. [25] designed a mass block in the form of split rings, which can obtain better acoustic performance through optimization of the distribution of eccentric masses. Chen et al. [26] proposed a new acoustic metamaterial comprised of two membranes and two ring masses which can effectively broaden the sound attenuation zone. Huang et al. [27] proposed two kinds of MAMs built by a polymeric membrane and a set of resonators based on the concept of bionic configuration philosophy, and this design greatly broadens the sound attenuation bandwidth in the lightweight condition. At present, most scholars select symmetric structures for the design of mass blocks, such as circle, ring, or symmetric distribution, which make the entire coupling system maintain a symmetric vibration behavior. The research on asymmetric masses or asymmetric MAMs has not been reported.

In this paper, an asymmetric mass block is firstly proposed, which is shaped as an eccentric ring. Based on Langfeldt’s analytical method [20], the STLs of MAMs with single-mass, double-mass, and four-mass are explored, respectively. The results are verified by the finite element model. Compared with MAMs with ordinary symmetric cylindrical masses, the superiority of the novel asymmetric MAMs in multi-peak and broadband sound insulation is proven. Furthermore, the influence of structural parameters such as mass eccentricity and distribution position on the sound insulation performance is discussed and the tunability of the sound insulation peak is proven. Finally, the Isight platform is used to optimize the structure of the asymmetric MAM with four masses. The main effect graphs and the Pareto graphs are drawn to analyze the contribution of structural parameters to sound insulation performance. The structure is optimized by the NSGA-Ⅱ algorithm, which greatly improved the sound insulation performance.

## 2. Model and Method

**Figure 1:**
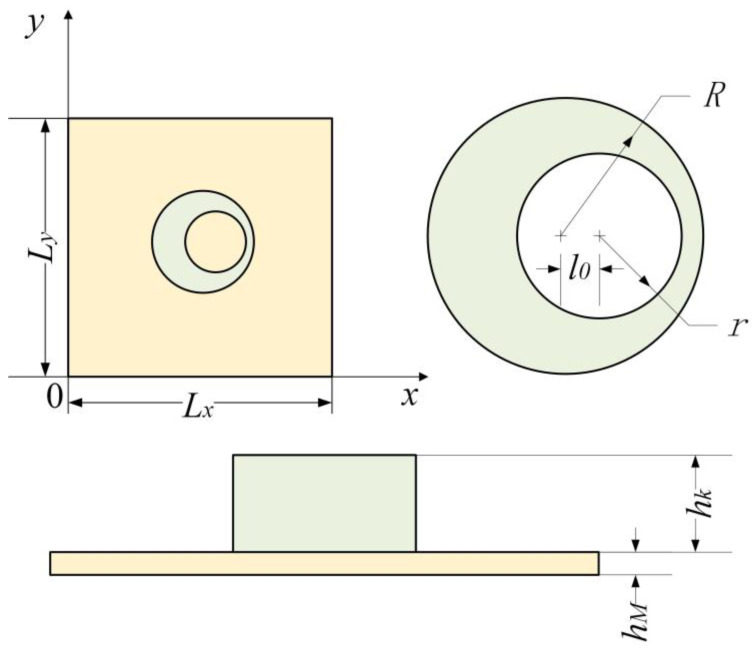
Definitions for the configuration of the MAM loaded with an asymmetric mass.

As shown in Figure 1, a coordinate system is set on a rectangular membrane whose thickness is *h_M_*, and the coordinate origin is located at one vertex of the rectangular membrane while the *x*-axis and *y*-axis coincide with both sides of the rectangle, whose lengths are *L_x_* and *L_y_*, respectively. The periphery of the membrane is fixed, and its surface density is *m_M_*. A uniform tension force per unit length, *T,* acts on the inside of the membrane. As shown in Figure 1, an asymmetric mass is loaded on the membrane. The geometric shape of this added mass is a cylinder, whose cross-sectional shape is an asymmetric ring, which is composed of a large circle minus a non-concentric small circle. The large circle is located at the center of the membrane with radius *R*, and the small circle, with radius *r*, is offset in the x-direction from the large one by *l*_0_. The height of the mass block is *h_k_*. A plane wave with amplitude of *P*_0_ is incident on the surface of the MAM. We used the analytical method presented by Langfeldt et al. [20] to analyze this MAM. The sound transmission loss (STL) of this MAM can be calculated according to the mass law:

(1) $$\mathrm{STL}=20\log_{10}\left|1+\frac{i\omega m^{\prime }}{2\rho_{0}c_{0}}\right|$$

where *ρ*_0_ and *c*_0_ are the density and speed of the medium through which the sound wave propagates, and m’ is the effective surface density of the membrane and can be calculated by:

(2) $$m^{\prime }=-\frac{T^{2}}{P_{0}\omega^{2}L_{x}^{2}\left[\begin{array}{cc} b^{T} & 0 \end{array}\right]X{\left(\Lambda -k^{2}I\right)}^{-1}{\left(X^{T}BX\right)}^{-1}X^{T}{\left[\begin{array}{cc} b & 0 \end{array}\right]}^{T}}$$

where **b** is the membrane excitation vector. **B** is a matrix in the standard eigenvalue equation: **Ax** = *k*^2^**Bx**, obtained from the equation system of this mass-loaded membrane [20]. The standard eigenvalue problem can be solved by the Arnoldi method [28] to obtain the eigenvalues and the eigenvectors of the coupled system. **Λ** is a diagonal matrix composed of the eigenvalues and **X** is a matrix in which the eigenvectors are arranged. **I** is the identity matrix.

## 3. Verification and Comparison

**Figure 2:**
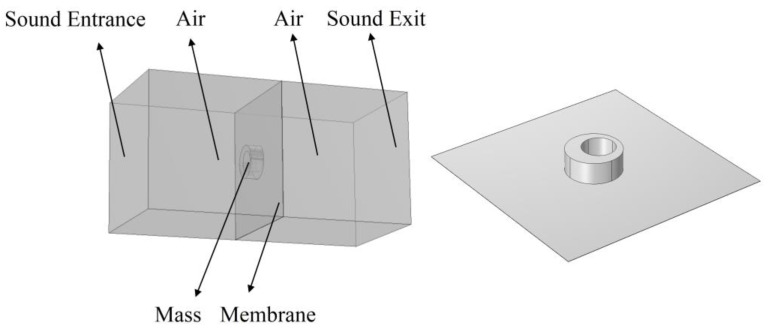
The 3D finite element model for the verification of the analytical model.

To verify the correctness of the analytical model, we compared the analytical calculation results of the STL with the results of the finite element model. In this paper, the commercial software COMSOL Multiphysics was used to establish a 3D finite element model, as shown in Figure 2, which consists of a membrane, a mass block, and two air domains. A plane wave with an amplitude of 1 Pa radiates outward from the sound entrance. After propagating through the first air domain, the plane wave interacts with the mass-loaded membrane, and part of the sound wave is transmitted to the second air domain. The calculation formula of sound transmission loss (STL) is:

(3) $$\mathrm{STL}=20\log_{10}\left(\frac{p_{\_in}}{p_{\_out}}\right)$$

where *p__in_* is the input sound pressure set as 1 Pa, and *p__out_* is the transmitted sound pressure, which can be obtained by calculating the average sound pressure of the sound exit boundary.

For the analytical model and the simulation model, polyetherimid (PEI), with density *ρ_M_* = 1270 kg/m^3^, Young’s modulus *E_M_* = 2.9 GPa, and Poisson’s ratio *ν_M_* = 0.44, was selected as the membrane material. The added mass material was steel, with density *ρ_k_* = 7860 kg/m^3^, Young’s modulus *E_k_* = 210 GPa, and Poisson’s ratio *ν_k_* = 0.3. The configuration was set with membrane length *L_x_* = *L_y_* = 20 mm, membrane thickness *h_M_* = 0.025 mm, large-circle radius *R* = 2.5 mm, small-circle radius *r* = 1.5 mm, small-circle offset *l_0_* = 0.5 mm, and mass height *h_k_* = 2 mm. The internal tension of the membrane was *T* = 120 N/m. In the calculation, standard atmospheric conditions were assumed, with *ρ_0_* = 1.225 kg/m^3^ and *c_0_* = 340 m/s. The calculation frequencies ranged from *f* = 50 to 1000 Hz.

**Figure 3:**
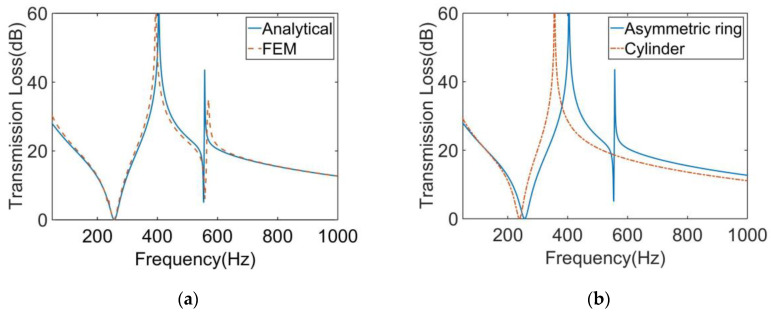
(**a**) Comparison of the analytical and numerical results for the STL curve of the MAM with an asymmetric ring. (**b**) Comparison of the MAMs with an asymmetric ring and a cylinder for the STL curve.

**Figure 4:**
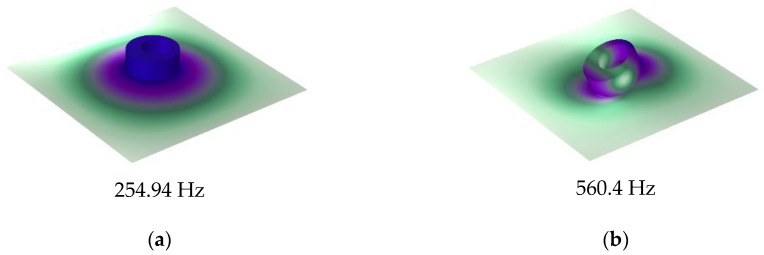
Eigenmodes of the single-mass-loaded membrane: (**a**) 254.94 Hz and (**b**) 560.4 Hz.

The calculation results of the finite element model and analytical model are shown in Figure 3a. It shows that the two results were in excellent agreement. The STL curve of this MAM presented two peaks located at near 400 Hz and 565 Hz. At each peak, there is only about 10 Hz deviation between the analytical results and the finite element results, which shows that the analytical method has high accuracy in predicting the STL. Figure 4 shows the two eigenmodes of this model at 254.94 Hz and 560.04 Hz, which can correspond well to the frequencies of STL valleys. From Figure 4, we can find that the first mode of the membrane is a symmetric mode. The average displacement of the membrane remained nonzero, which led to the appearance of the first valley. The unconventionality of this MAM is reflected in the second mode of the membrane, which shows an asymmetric pattern. This special mode also had large average displacement and is the reason why the second STL valley exists. There was no eigenmode at the peak, but the peak was also directly affected by the eigenmode because the vibration form at the peak is the superposition of several eigenmodes, so that the surface displacement directions of two parts of the membrane were opposite, which is the optical vibration mode. The average displacement was about zero, which means the sound was well-blocked.

We also calculated the STL of the MAM loaded with traditional cylindrical masses which have the same weight as the asymmetric one. The height and cross-sectional area of the two mass blocks were equal. The results are compared as shown in Figure 3b. It can be seen from the figure that the main peak of the asymmetric model shifted backward by about 50 Hz compared with the traditional model. The more obvious difference is that only the asymmetric one produced a second peak, and it had a higher STL value in a wider frequency range than the traditional MAM.

**Figure 5:**
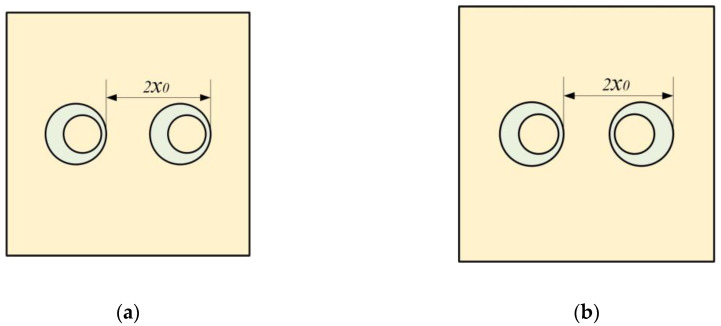
Definitions for the configuration of the MAM loaded with two masses: (**a**) asymmetric arrangement and (**b**) symmetric arrangement.

**Figure 6:**
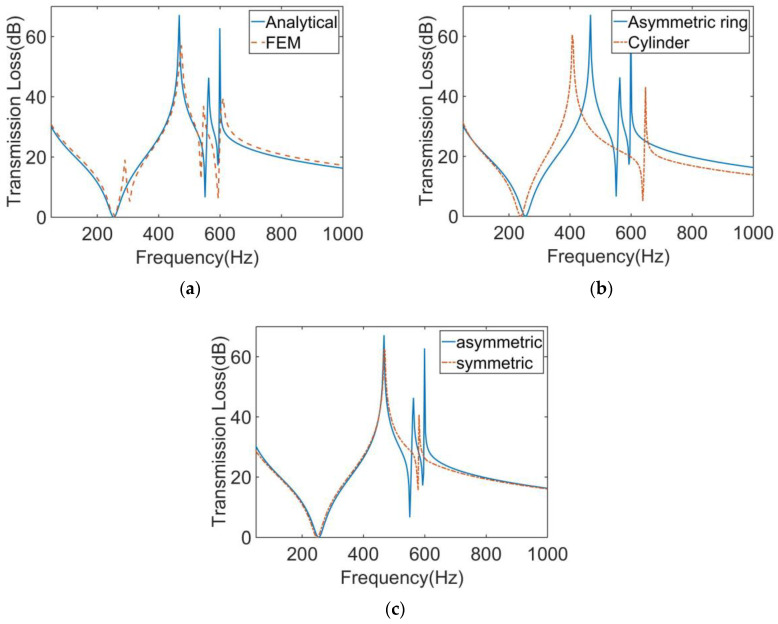
(**a**) Comparison of the analytical and numerical results for the STL curve of the MAM with two asymmetric rings. (**b**) Comparison of the MAMs with two asymmetric rings and two cylinders for the STL curve. (**c**) Comparison of the MAMs with two asymmetric rings, asymmetrically and symmetrically arranged.

**Figure 7:**
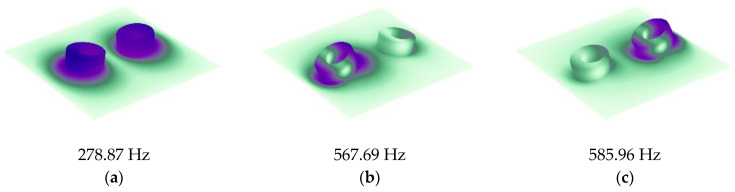
Eigenmodes of the double-mass-loaded membrane: (**a**) 278.87 Hz, (**b**) 567.69 Hz, and (**c**) 585.96 Hz.

Based on the single-mass model, we further calculated the STL of MAM containing multiple masses. As shown in Figure 5a, two mass blocks were distributed on the membrane with *x*_0_ = 5 mm, whose eccentric directions were the same. The distribution position was symmetrical, but due to the asymmetry of the mass, the whole structure was asymmetric to the *y*-axis. Figure 6a shows the STL curve calculated by the finite element method and the analytical method. As can be seen from the figure, both the analytical result and the finite element result can reflect three large peaks. In the finite element results, there was a small sound insulation peak at about 300 Hz, which could not be found in the analytical result. It is speculated that there are two modes with similar frequencies at this position, but the analytical model cannot accurately identify them, so a peak is ignored. This mode is presumed to be caused by the eccentric arrangement of the mass blocks, because there is no such special peak in the FEM model of any MAM with centrally arranged mass blocks. However, this small peak did not significantly affect the sound insulation performance of the MAM, so we will ignore it in subsequent discussions and consider the analytical model to be practical in most cases. Figure 7 shows three eigenmodes of this model at 278.87 Hz, 567.69 Hz, and 585.96 Hz, which were in good agreement with the frequencies of the STL valleys. It can be seen from Figure 7 that the first mode exhibited symmetry of vibration while the other two exhibited asymmetry, and all three modes caused the MAM to generate STL valleys.

We also compared this result with that of the MAM with two cylinders, as shown in Figure 6b. It can be seen that, compared with the traditional cylindrical type, the main peak of the asymmetric one shifted backward by about 70 Hz, and the two sub-peaks were closer to the main peak, forming an excellent sound insulation band. The traditional MAM has only one sub-peak. Since the structure can be regarded as the coupling of two single-mass models, the two modes generated by each independent structure are completely symmetrical and their frequencies are close, so that only one new peak was excited. On the contrary, the asymmetry is why the novel MAMs had two sub-peaks. To further discover the difference between symmetric and asymmetric MAMs, we rotated one of the mass blocks 180°, as shown in Figure 5b, meaning that the two mass blocks formed a symmetric arrangement. Therefore, the MAM returned to a symmetric state, and we compared the STL curves of the two MAMs, as shown in the Figure 6c. We found that when the MAM was symmetric, only one sub-peak appeared. This phenomenon is easily explained by the fact that the symmetric modes produced by the two masses had the same frequency. This further demonstrates the unique advantages of the asymmetric MAM.

**Figure 8:**
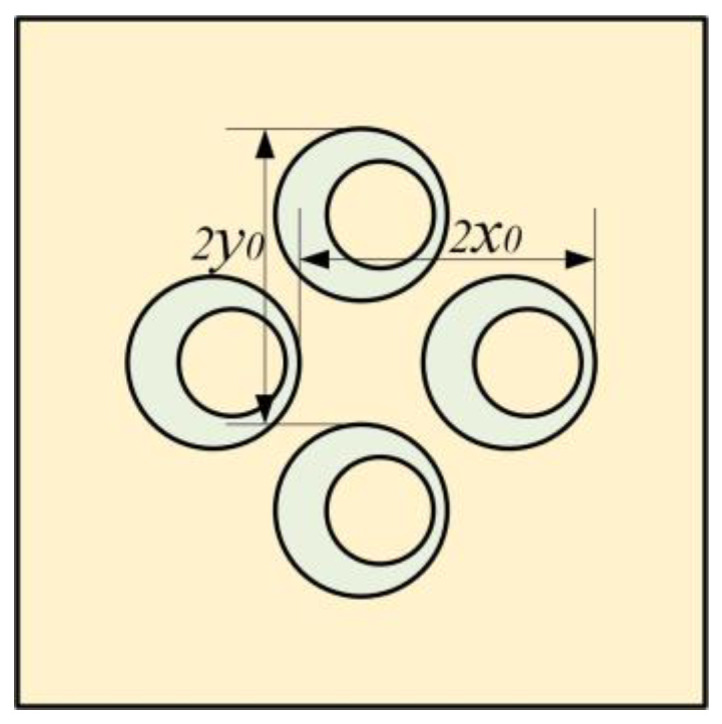
Definitions for the configuration of the MAM loaded with four asymmetric masses.

**Figure 9:**
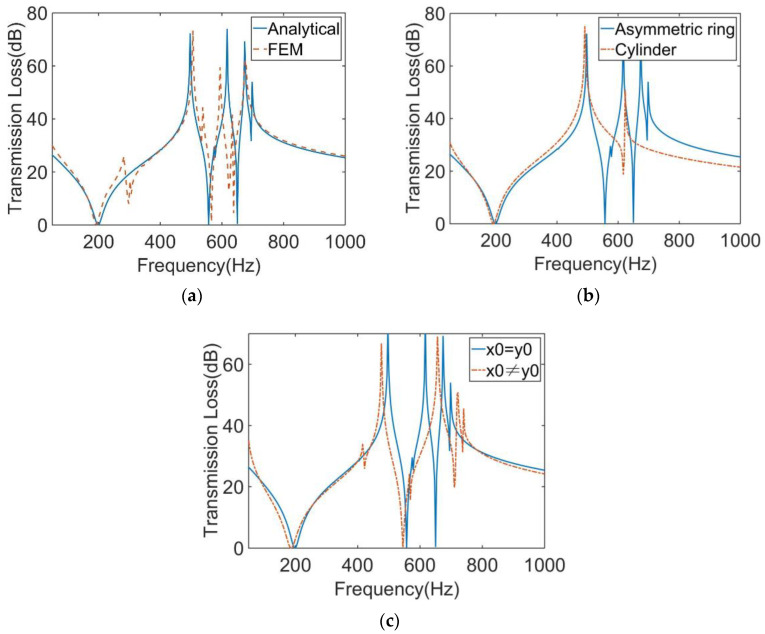
(**a**) Comparison of the analytical and numerical results for the STL curve of the MAM with four asymmetric rings. (**b**) Comparison of the MAMs with four asymmetric rings and four cylinders for the STL curve. (**c**) Comparison of the MAMs with mass positions x_0_ = y_0_ and x_0_ ≠ y_0_ for the STL curve.

**Figure 10:**
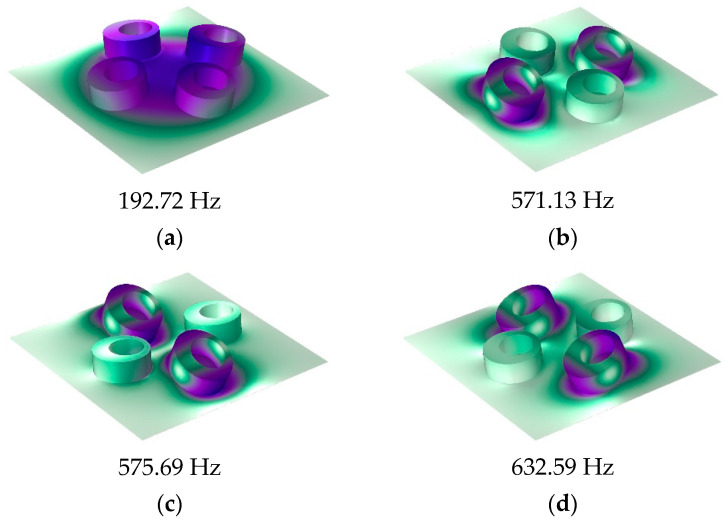

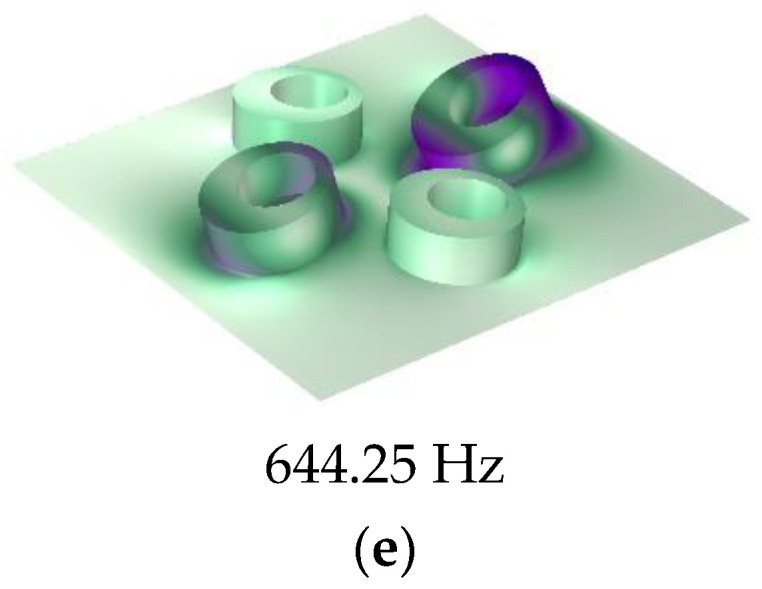
Eigenmodes of the four-mass-loaded membrane: (**a**) 192.72 Hz, (**b**) 571.13 Hz, (**c**) 575.69 Hz, (**d**) 632.59 Hz, and (**e**) 644.25 Hz.

Figure 8 shows the structure of the MAM with four asymmetric rings which have the same eccentric directions. They are distributed on the membrane symmetrically with *x*_0_ = 5 mm and *y*_0_ = 5 mm. Figure 9a shows the finite element and analytical calculation results, and they were in excellent agreement in most frequency bands. The four-mass model has similarity to the two-mass model in that there are two masses distributed in the y-direction in the same way as those distributed in the x-direction. Due to the orthogonal symmetry of the square membrane, the effects of the two groups of masses on the membrane were also similar, resulting in a set of orthogonally symmetric modes, in the form of strengthening the anti-resonance modes. Therefore, the number of obvious sound insulation peaks did not change (only a new small peak appeared, but it is not discussed in this paper and we consider that it only has two obvious sub-peaks), but the two sub-peaks have been significantly improved and widened. There was also a small peak at about 300 Hz in the FEM model, which did not exist in the analytical model. This is for the same reason as the two-mass model, and we also chose to ignore it. Figure 10 shows the five eigenmodes of this MAM. It can obviously be found that the second and third eigenmodes were close in frequency and had orthogonal symmetry in shape. This phenomenon is also reflected in the fourth and fifth eigenmodes. In addition, compared with the two-mass model, the three obvious peaks were shifted to high frequencies when the four-mass model was added. The reason for this phenomenon is that the masses and the elastic force of the membrane formed a spring oscillator system, whose resonant frequency increased when the added mass increased. 

Figure 9b shows the comparison between the traditional MAM loaded with four cylinders and the MAM with four asymmetric rings. The traditional MAM has only one sub-peak. Since the square membrane has orthogonal symmetry, the new modes caused by coupling between the four cylindrical masses and the membrane had the same frequency. The novel MAM is better than the traditional MAM for sound insulation in a larger frequency range and has more peaks, so the novel MAM has higher sound insulation performance for both broadband and specific frequency band. Furthermore, we studied the case of *x_0_* ≠ *y_0_*. We left *x*_0_ = 5 mm unchanged and changed *y*_0_ = 4 mm to draw the STL plot, as shown in Figure 9c. As can be seen from the figure, the number of distinct peaks did not change. This phenomenon is normal because in this form of MAM, one mass block in the x-direction and one mass block in the y-direction can also be regarded as a group, thus forming an oblique line symmetric structure. Therefore, the shape of the STL curve was similar to that when *x*_0_ = *y*_0_. However, the frequency of each peak has changed. Compared with the case of *x*_0_ *= y*_0_, the main peak has moved forward, while the two sub-peaks have moved backward.

## 4. Discussion

**Figure 11:**
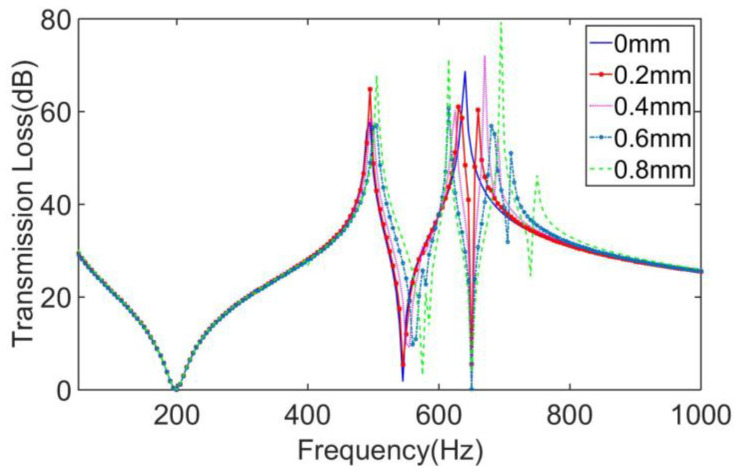
Comparison of the MAMs with different mass eccentricities.

In order to investigate the influence of structural parameters on the sound insulation performance of this MAM, we have performed further work: The STLs of the four-mass MAMs with mass eccentricity *l*_0_ = 0 mm, 0.2 mm, 0.4 mm, 0.6 mm, and 0.8 mm were calculated, respectively, as shown in Figure 11. It can be found from the figure that when *l*_0_ = 0 mm, which means the masses are completely centrosymmetric rings, the STL curve showed two large peaks at around 500 Hz and 640 Hz, and two valleys at around 200 Hz and 550 Hz, which is in line with the characteristics of the symmetrical MAMs. As *l*_0_ gradually increased, the frequency of the first valley did not significantly change, and the positions of the first peak and the second valley only underwent a small backward shift. The main changes were concentrated at the second peak. With the emergence of the asymmetry of the mass block, the original second peak was split into two, and with the increase of *l*_0_, the two new peaks showed a tendency to offset away from each other. The main reason for the frequency shift comes from the change of the position of the mass centroid, which caused the distribution of the loading force of the mass to change, so the whole coupling structure produced different modes. This shows that by increasing the eccentricity, *l*_0_, the distribution distance of the two secondary peaks can be made farther, which is an effective way to regulate the sub-peaks.

**Figure 12:**
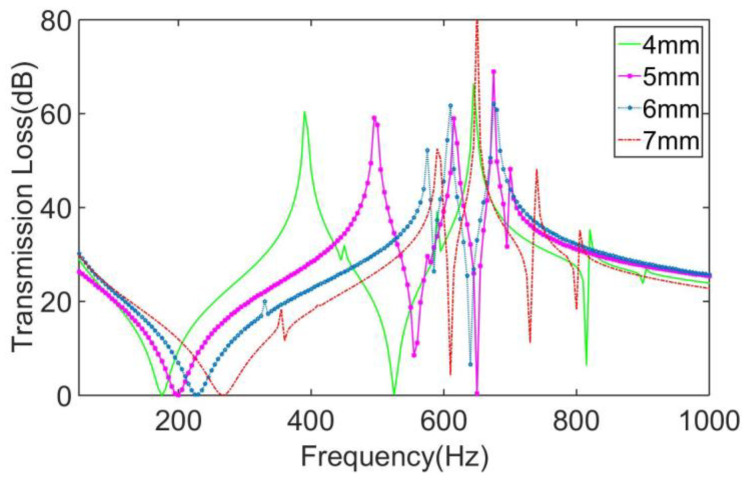
Comparison of the MAMs with different mass positions.

The STLs of the MAMs with the distribution position *x*_0_ = *y*_0_ = 4 mm, 5 mm, 6 mm, and 7 mm are calculated, respectively,, as shown in Figure 12. It can be seen that with the change of the distribution position, all the peaks and valleys of the MAMs have significantly changed. According to Figure 12, the following conclusions can be drawn: With the increase of *x*_0_ and *y*_0_, the first and second valleys and the first peaks all tended to shift backwards, and the distance between the two sub-peaks relative to the main peak generally tended to decrease. The shift of the peaks was also caused by the change of the position of the loading force, but to a larger extent, so the shift was more obvious. It is concluded that the distribution position can be used as a means to tune the main peak of metamaterials. Combined with the conclusion in the above section, by matching the eccentricity with the distribution position of the asymmetric masses, it is feasible to regulate the overall sound insulation performance of the MAM and design it for specific usage needs.

## 5. Optimization

In the section above, we analyzed the effect of the parameters *l_0_* and *x_0_* on the STL performance of the MAM. However, the influence of other structural parameters on sound insulation characteristics has not been explored. Furthermore, we hoped to find a set of optimized structural parameters that can maximize the performance of this MAM. Therefore, we used Isight software for optimization analysis. The Isight platform can integrate a variety of software for engineering optimization problems [29,30,31]. For the structure of this MAM, we selected the membrane thickness, *h_M_*, the membrane side length, *L_x_* (*L_x_ = L_y_*), the mass external radius, *R* (*r* = 0.6 *R*), the mass height, *h_k_*, the mass eccentricity, *l_0_*, the mass position, *x*_0_ (*x*_0_ = *y*_0_), and internal tension, *T,* as design variables. Their value ranges were 0.02 mm ≤ *h_M_* ≤ 0.03 mm, 15 mm ≤ *L_x_* ≤ 30 mm, 2 mm ≤ *R*≤3 mm, 1.5 mm ≤ *h_k_* ≤ 3 mm, 0 ≤ *l*_0_ ≤ 1 mm, 4 mm ≤ *x*_0_ ≤ 8 mm, and 90 N/m ≤ *T* ≤ 150 N/m. We attached importance to the sound insulation performance of low-frequency broadband and lightweight conditions of the MAM, so we took the average STL in the calculated frequency band, the frequency bandwidth higher than 30 dB, and the average surface mass density of MAM as the optimization objectives. They are defined as *TLs*, *frq_30_*, and *m,* respectively. Therefore, the multi-objective optimization problem can be described as:

(4) $$\begin{array}{l} \max\;\;TLs,\;\;frq_{30}\\ \min\;\;m\\ s.t.\;\;\;0.02\;\mathrm{mm}\leq h_{M}\leq 0.03\mathrm{mm}\\ \;\;15\;\mathrm{mm}\leq L_{x}\leq 30\;\mathrm{mm}\\ \;\;2\;\mathrm{mm}\leq R\leq 3\;\mathrm{mm}\\ \;\;1.5\;\mathrm{mm}\leq h_{k}\leq 3\;\mathrm{mm}\\ \;\;0\leq l_{0}\leq 1\;\mathrm{mm}\\ \;\;4\;\mathrm{mm}\leq x_{0}\leq 8\;\mathrm{mm}\\ \;\;90\;N/m\leq T\leq 150\;N/m \end{array}$$

**Figure 13:**
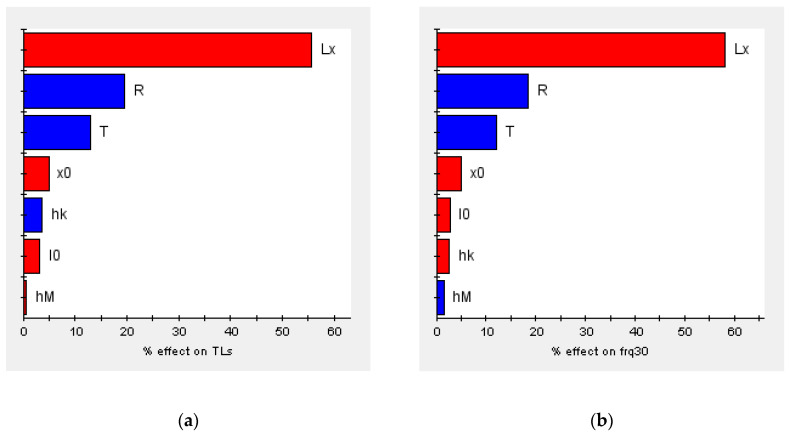
Pareto graphs of the optimization objectives: (**a**) *TLs* and (**b**) *frq_30_*.

**Figure 14:**
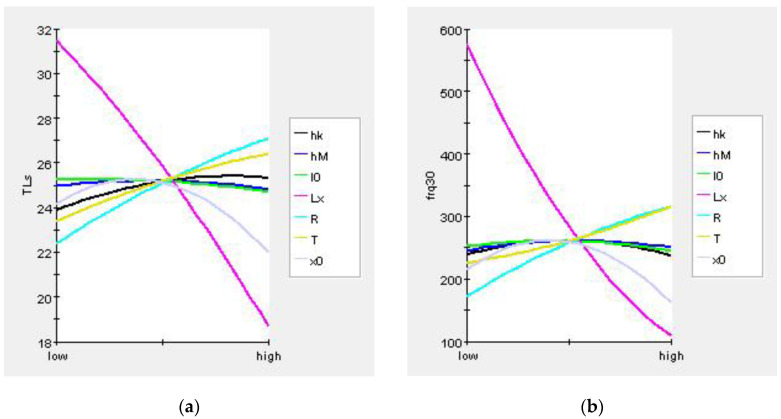
Main effect graphs of the optimization objectives: (**a**) *TLs* and (**b**) *frq_30_*.

The general optimization steps were design of experiments (DOE), establishment of approximate models, and the optimization algorithm. DOE is a mathematical statistical method to study the influence of design variables on optimization objectives. Since the sampling points in the design space are infinite, a specified method or rule is required to select the limited sampling points. The Latin hypercube design can achieve uniform and continuous sampling in the specified area. Its principle is that in the *N*-dimensional space composed of *N* factors, within the coordinate interval [*x_Kmin_*, *x_Kmax_*] of the *K*(*K*∈[1, *N*])-dimension, the *K*-dimensional space is divided into *M* intervals, each cell is recorded as [*x_Ki-1_*, *x_Ki_*], and then a sample point is randomly selected from each intervals, so a total of *M* sample points is selected. The optimal Latin hypercube design is an improvement on the basis of the Latin hypercube design, which enhances the space-filling ability and the uniformity of samples. Therefore, the optimal Latin hypercube was used as the DOE method in this paper. In the design variable space, 150 groups of sample variables were selected for calculation. The pareto graphs and main effect graphs obtained by statistics according to the sample calculation results are shown in Figure 13 and Figure 14. These two kinds of graphs represent the ways in which design variables affect the objective function. In the Pareto graphs, the size of the bar indicates the percentage contribution of the design variable to the influence of the objective function, where blue indicates a positive effect and red indicates a negative effect. It can be found from the figure that *L_x_*, *T,* and *R* had obvious effects on both *TLs* and *frq_30_*, while the others had weak effects. The meaning of the main effect graph representation is similar to that of the Pareto graph, which can show how the objective functions changed with the design variables. Obviously, the larger the absolute value of the slope of the curve, the greater the influence of the design variables on the objective functions. Therefore, the same conclusion can be drawn from the main effects graphs.

**Figure 15:**
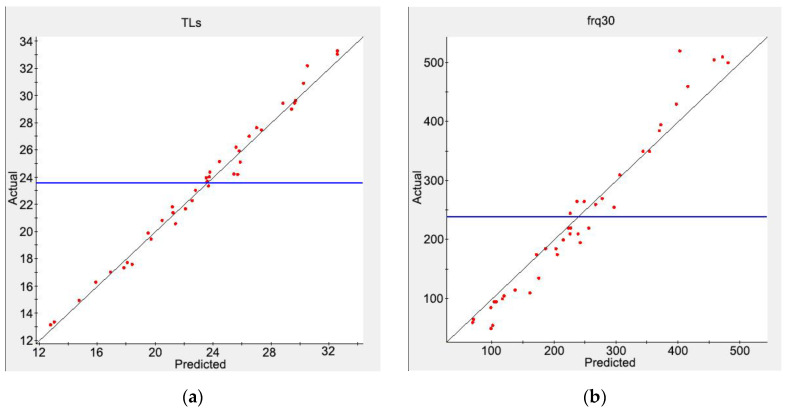
Scatter plots of the approximate models: (**a**) *TLs* and (**b**) *frq_30_*.

After the DOE, an approximate model was established to assist in the analysis. The purpose of establishing the approximate model was to find the relationship between design variables and optimization objectives, so that the calculation time and optimization cost can be greatly reduced when the optimization calculation is carried out. The principle for the approximate model is as follows:

(5) $$Y\left(x\right)=\tilde{Y}\left(x\right)+\varepsilon$$

where Y(x) is the actual response value of the objective function, $\tilde{Y}(x)$ is the approximate value of the objective function response, and *ε* represents the error between the actual value and the approximate value and is usually a normal distribution. The proximity between the approximate model and the actual situation was evaluated by R-squared (*R*^2^), and the closer *R*^2^ is to 1, the higher the accuracy and credibility of the approximate model. The response surface model (RSM), radial basis function (RBF) neural network model, orthogonal polynomial model, and the Kriging model are commonly used approximate models. Combined with the DOE data above, the four models were all used to construct an approximate model of the objective functions. Here, 40 groups of data from the DOE samples were randomly selected for error analysis. The values of the model accuracy parameter *R*^2^ are shown in Table 1. An approximation model can be considered as credible if the *R*^2^ value is greater than 0.9, so these approximation models all met the requirements. Among them, the RSM model had the best approximate effect on *TLs*, and the Kriging model had the best approximate effect on *freq_30_*. Therefore, these two methods were selected to establish approximate models for the two optimization objectives for the subsequent optimization calculation. Figure 15 shows the scatter plot of the actual value and the predicted value of the approximate models. The black line is the contour line, and the blue line is the actual average. Most of the points in the figure are close to the contour line, which also indicates that the approximate models can meet the requirements.

**Figure 16:**
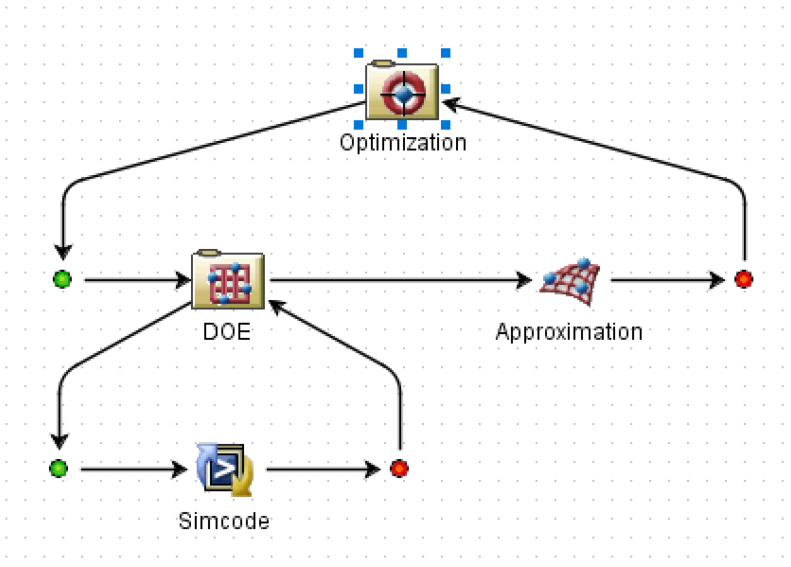
Schematic of the optimization process.

**Figure 17:**
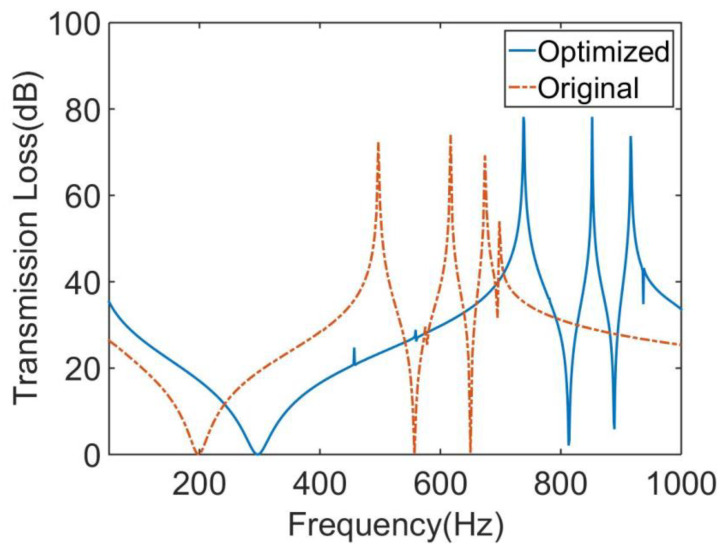
Comparison of the original and optimized MAMs for the STL curve.

The genetic algorithm is a global search optimization algorithm abstracted from Darwin’s law of evolution, which has a strong global solution ability. The non-dominated sorting genetic algorithm II (NSGA-Ⅱ) is a new type of genetic algorithm. It has three characteristics: fast non-dominated sorting approach, fast crowded distance estimation procedure, and simple crowded comparison operator [32]. It is a widely used multi-objective optimization algorithm. In this paper, NSGA-Ⅱ was used to solve the multi-objective optimization problem combined with the approximate model established above. Figure 16 shows the optimization model established by the Isight platform, in which the Simocode module was used to call the analysis software. The MAM design variable and optimization objective values after optimization were compared to original values, as shown in Table 2 and Table 3, respectively. A comparison of STL curves before and after optimization is shown in Figure 17. As can be seen in Table 3, the MAM’s average sound transmission loss and frequency bandwidth above 30 dB were significantly improved after optimization: 15.7% and 11.5%, respectively. This means that the broadband sound insulation capability of the new MAM has been enhanced. Meanwhile, the optimized surface mass density also decreased by 30.01%, which made the MAM more lightweight and compact. This shows that the optimization method in this paper can indeed improve the MAM performance in all aspects. However, it can be found from Figure 17 that the optimized STL curve had a significant change compared with the original one: the overall peak value moved to a high frequency, which makes the sound insulation capability in the frequency band seem to be distributed less evenly because this is not considered in the optimization problem. If we want to make the STL distribution more uniform or improve the sound insulation effect in a small frequency band, we need to redesign the objective function. This is worth studying, and we are considering it as our next focus.

## 6. Conclusions

In this paper, a novel MAM with asymmetric masses was firstly proposed for low-frequency broadband sound insulation, where asymmetry was achieved by constructing a structure of an eccentric ring. The sound insulation characteristics were studied by the analytical method and the finite element model. It was found that the novel MAM was better in sound insulation in a wider frequency range compared with the traditional MAM with equal mass. Moreover, taking the four-mass model as an example, the influence of structural parameters on its performance was studied. Finally, the Isight software was used to optimize the MAM. After optimization, the average STL of the MAM was improved by 15.7%, the bandwidth above 30 dB was improved by 11.5%, and the mass density was reduced by 30.01%. The optimization results show that the optimization method in this paper is feasible, but it still involves simplification. Therefore, a more comprehensive optimization problem is necessary to become the next research focus.

## Figures and Tables

**Table 1:** *R*^2^ values of the four approximate models of the optimization objectives.

	RSM	RBF	Orthogonal	Kriging
*TLs*	0.98627	0.98303	0.984588	0.91226
*frq*_30_	0.90442	0.90444	0.90559	0.93409

**Table 2:** Original and optimized design variable values.

Variables	Original Values	Optimized Values
*L_x_*	20 mm	17.8635 mm
*h_M_*	0.025 mm	0.02386 mm
*R*	2.5 mm	2.1138 mm
*h_k_*	2 mm	1.5534 mm
*T*	120 N	148.6532 N
*x*_0_	5 mm	4.5826 mm
*l*_0_	0.5 mm	0.6565 mm

**Table 3:** Original and optimized optimization objective values.

Optimization Objectives	Original Values	Optimized Values
*TLs*	23.334 dB	26.997 dB
*frq*_30_	338 Hz	377 Hz
*m*	2.006 kg/m^2^	1.404 kg/m^2^

## Data Availability

The data used to support the findings of this study are included within the article.
